# Uterine giant cell tumor of soft tissue: A case report

**DOI:** 10.1097/MD.0000000000035414

**Published:** 2023-10-20

**Authors:** Yao Jiang, Kaiyan Chen, Mengting Yu, Juan Qin, Juntao Wang

**Affiliations:** a Guizhou Medical University, China; b The Maternal and Child Health Care Hospital of Guizhou Medical University, China.

**Keywords:** case report, chemotherapy, giant cell tumors, soft tissue neoplasms, surgery, uterine neoplasms

## Abstract

**Introduction:**

Giant cell tumor of soft tissue (GCT-ST) is a rare primary soft tissue tumor. GCT-ST mainly occurs in the trunk and extremities. There is no standard treatment for GCT-ST. This paper reports a rare case of primary uterine GCT-ST.

**Case presentation:**

A 48-year-old female patient underwent a transabdominal subhysterectomy for uterine leiomyoma. Postoperative pathological examination showed GCT-ST with unclear tissue boundary (10.0 × 6.0 × 5.0 cm). A small amount of GCT-ST tissue could be seen on the local edge of the leiomyoma. Residual tumor tissue was found around the uterine cavity. The patient reported persistent lower abdominal distension pain 3 months after the operation. Pelvic and abdominal imaging showed a huge tumor and multiple pelvic and abdominal organ metastasis. No pulmonary metastasis was found. Exploratory surgery revealed widespread metastases in the abdominal and peritoneal cavities, involving both ovaries, right tubal serous membrane, appendix serous membrane, bladder, pelvic peritoneum, and abdominal wall incision. After surgery, the patient had 6 cycles of docetaxel and carboplatin but stopped treatments due to economic reasons. The patient died 3 months later because of multiple organs failure.

**Conclusion:**

GCT-ST is generally benign but has unpredictable behavior. A massive recurrence with wide invasion is possible after subtotal resection.

## 1. Introduction

Giant-cell tumor (GCT) of soft tissues (GCT-ST) is a rare primary soft tissue tumor. GCT-ST has unpredictable biological behavior; most are benign but may sometimes show aggressiveness, invasiveness, local recurrences, and rare distant metastasis. GCT-ST shares some clinical characteristics with GCT of bone but differs in immunophenotype and heredity.^[1,2]^ GCT-ST can occur in patients of any age. Tumors often occur in the extremities and torso, especially the thigh.^[3–5]^

In general, the tumor is generally composed of solid components with a small amount of cystic components. The boundary is often clear. Under the microscope, the cells with round or oval vesicular nucleus form multinodular aggregates, a large number of osteoclast-like multinucleated giant cells with obvious atypia, and the tumor stroma is rich in blood vessels.^[2]^

GCT-ST is rare, and its treatment lacks uniformity. At present, complete surgical resection is the most accepted treatment, but there is controversy regarding the surgical margin. We report an extremely rare case of uterine GCT-ST.

## 2. Case presentation

**Figure 1.**
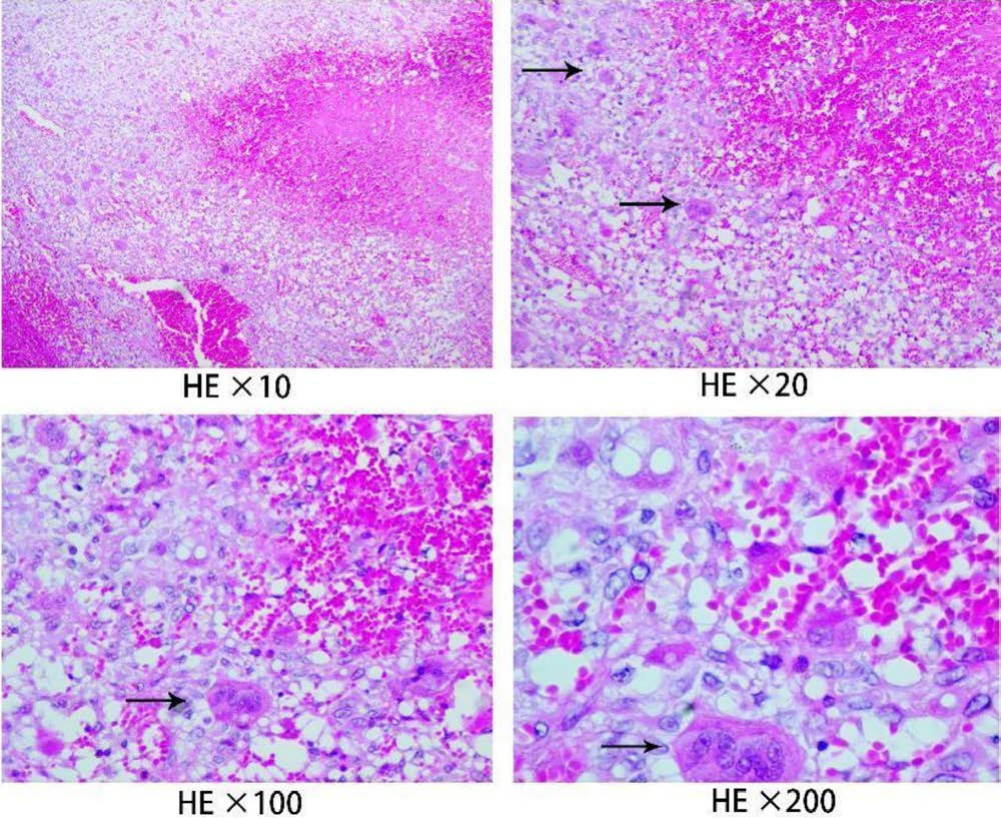
Hematoxylin & eosin staining showing multinodular aggregates formed by round or fusiform tumor cells. The nuclei were mostly round or oval and sometimes fat fusiform, which were vesicular nuclei. Osteoclast-like giant cells were uniformly distributed in single cells, and the nuclei were the same as monocytes.

A 48-year-old woman with a 1-month history of persistent lower abdominal distension pain was admitted on December 21, 2017. The patient had hypertension for 8 years and an allergy to cephalosporin. The patient underwent a hysterectomy for uterine fibroids in August 2017. In this Study, all human subjects provided written informed consents with guarantees of confidentiality. Postoperative pathology showed a small amount of GCT-ST components on the local edge of uterine leiomyoma, and the free broken tissue was tumor cells, with a total volume of 10.0 × 6.0 × 5.0 cm, with large areas of necrosis and tumor tissue residues around the uterine cavity (Fig. 1). Immunohistochemistry showed a few mononuclear cells were positive for CD and SMA. All mononuclear and multinucleated cells were positive for vimentin. CD68 was positive in multinucleated cells. Ki-67 accounted for 10%–20% of mononuclear cells. P53 accounted for 20%–30% of mononuclear cells. P63 was positive in some osteoclast-like giant cells. The lesion was confirmed as GCT-ST.

The patient reported persistent but tolerable lower abdominal distension pain 1 month after surgery (September 2017). The patient did not receive treatment.

**Figure 2.**
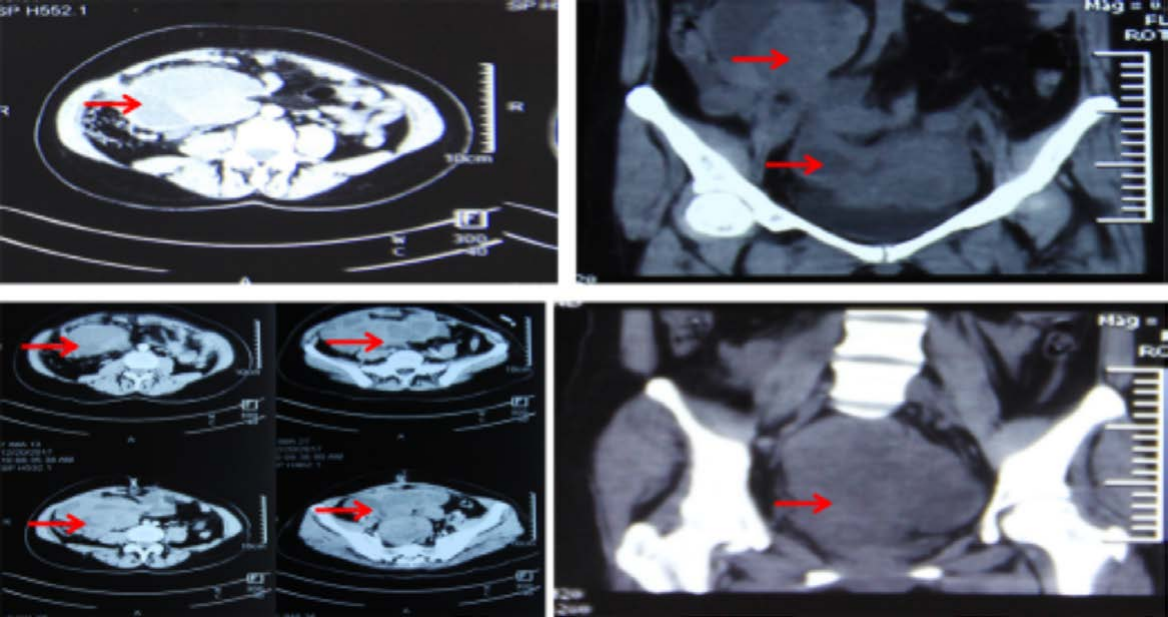
Pelvic contrast-enhanced computed tomography shows multiple cystic spaces occupying lesions in the pelvis, changes after hysterectomy, and abnormal enhancement of soft tissue nodules in the incision (December 2017).

**Figure 3.**
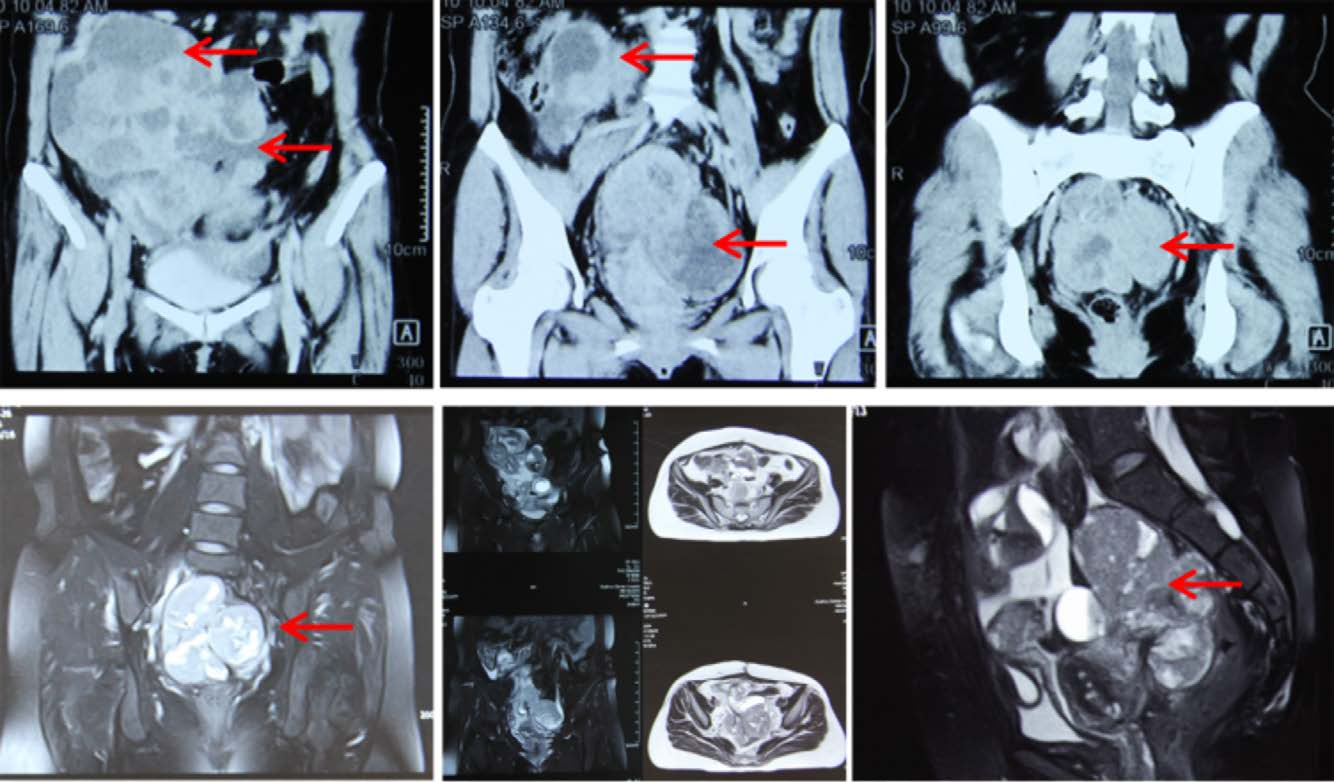
Abdominal magnetic resonance imaging showed a huge cystic-solid space from the right lower abdomen to the pelvis, with intratumoral hemorrhage, suspicious abdominal dissemination, and suspicious malignant lesions (December 2017).

**Figure 4.**
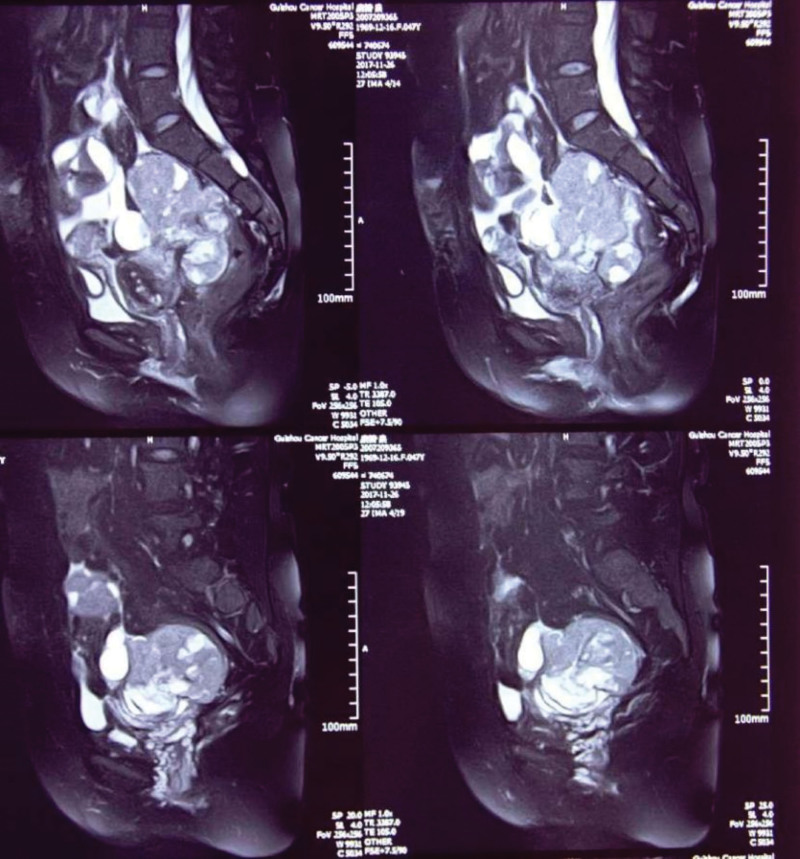
Pelvic magnetic resonance imaging showed a huge cystic-solid space from the pelvis to the lower abdomen, with intratumoral necrosis, hemorrhage, and cystic degeneration, and multiple space-occupying shadows on both sides of the incision after surgery on the anterior abdominal wall of the lower abdomen (December 2017).

Outpatient B-mode ultrasound (December 2017) showed subtotal hysterectomy, and multiple mixed echo masses (104 × 49 mm) and cystic masses were seen in the pelvis. A gynecological examination showed a 15-cm mass palpated on the right side of the pelvic cavity, with an irregular shape, moderate quality, tenderness, poor mobility, and unclear boundary. Pelvic contrast-enhanced computed tomography (CT) showed multiple cystic lesions in the pelvis, hysterectomy changes, and abnormal enhancement of soft tissue nodules in the incision (Fig. 2). Abdominal magnetic resonance imaging (MRI) showed a huge suspicious malignant cystic-solid lesion in the right lower abdomen and pelvis, with intratumoral hemorrhage and suspicious abdominal dissemination (Fig. 3). Pelvic MRI showed a huge cystic-solid space from the pelvis to the lower abdomen, with intratumoral necrosis, hemorrhage, cystic degeneration, and multiple space-occupying shadows on both sides of the incision after surgery on the anterior abdominal wall of the lower abdomen (Fig. 4). Chest CT showed suspicious lesions in the right lung. The tumor marker CA125 was significantly increased.

Open lesion resection was performed under general anesthesia. A mass (50 × 40 × 40 mm) was found subcutaneously in the original incision of the lower abdomen during the operation, and fish-like tissue was found in the mass. Exploration showed no ascites in the pelvic and abdominal cavities, and several cystic and solid masses of different sizes (50–120 mm) were found in the pelvic and abdominal cavities. On the surface of the bladder, the intestine and peritoneum were scattered with peanut- to millet-size nodules, and millet-size lesions were seen at the edge of the spleen. Scattered implantation lesions were found in the pelvic peritoneum. The lesions in the pelvic and abdominal cavity extensively adhered to the sigmoid colon, ascending colon, rectum, and bladder, invading the serous layer of the sigmoid colon and ascending colon, the rectal seromuscular layer, and the muscular layer of the bladder. When the electric knife touched the adhesion, the lesion ruptured, with a fish-like texture and dark brown/red liquid.

Postoperative pathological examination showed GCT-ST involvement in both ovaries, right tubal serous membrane, appendix serous membrane, bladder, pelvic peritoneum, and abdominal wall incision. No lymph node metastasis was found. The patients received adjuvant docetaxel (120 mg the first time, average dose 75 mg/m^2^) combined with carboplatin (500 mg, mean dose 314 mg/m^2^), planned for 9 cycles.

The patient finally received 6 cycles of chemotherapy and stopped due to economic reasons. The patient did not receive other treatments and died in April 2018 from multiple organ failure.

## 3. Discussion

GCT-ST is a rare primary soft tissue tumor. GCT-ST mainly occurs in the trunk and extremities. There is no standard treatment for GCT-ST. This paper reports a rare case of primary uterine GCT-ST. The take-home message of this case is that even if GCT-ST is generally benign, it can have unpredictable behavior. A massive recurrence with widespread invasion is possible after subtotal resection in a non-oncological context.

According to the World Health Organization (WHO) classification, GCT-ST belongs to the intermediate (critical) malignant fibrous histiocytic tumor category.^[6,7]^ Unlike malignant GCT-ST, GCT-ST is characterized by a lack of or little cellular atypia, low to moderate mitotic activity, and no atypical characteristics.^[6]^ Malignant GCT-ST may have atypia and necrosis. In malignant GCT-ST, histological evidence shows that sarcoma-like changes have occurred in cellular and nuclear pleomorphism. Benign GCT-ST lesions lack atypical, pleomorphic, and atypical mitosis.

The tumor in this patient showed locally invasive biological behavior. GCT-ST is most common in the superficial soft tissue of the extremities but can involve deep soft tissues. Still, organs are rarely affected. Whether there is a relationship between the rare location of the tumor and the malignant degree of the tumor is not reported in the literature. In osteosarcoma, the prognosis of tumors in the spine or pelvis is worse than in extremities,^[8]^ and a similar tendency might be observed in GCT-ST. GCT-ST can transform into a secondary malignant tumor after radiotherapy (RT) or recurrence,^[9]^ which would support that this patient progressed rapidly after the operation. Because of the benign course of GCT-ST, some authors suggested that when the surgical margin is clear, and the mass is fully removed, the recurrence rate is very low, but there is usually local recurrence or even metastasis after incomplete surgical resection.^[10]^ Still, the invasive growth patterns may involve deep and fine anatomical structures, such as neurovascular bundles, which make complete surgical resection difficult.^[11]^

Because GCT-ST is rare, beyond a suggestion of complete surgical removal, there is no consensus regarding its management. This patient did not receive further treatment after the initial diagnosis, but a margin revision should have been performed. In addition, the role of chemotherapy in GCT is not clear. It has been reported that combination chemotherapy has some effect on metastatic invasive uterine GCT-ST treated with liposomal doxorubicin, ifosfamide, and bevacizumab.^[12]^ Therefore, chemotherapy selection might play an important role in the response. The case reported here received docetaxel and carboplatin, but the patient died 3 months after the last cycle of chemotherapy. Because the chemotherapy regimen was not completed (6 on 9 cycles), the effectiveness of the chemotherapy regimen of the patient cannot be evaluated. Still, there are no treatment guidelines for GCT-ST. When the location of the tumor rules out the possibility of resection,^[3]^ it is necessary to seek other treatments. For those unresectable GCT-ST, because there are few reports and limited data related to treatment, there is no exact treatment plan at present. The treatment of GCT-ST is mainly guided by the reported cases and treatment choices extrapolated from the treatment of bone GCT.^[13]^ In a case report of an unresectable tumor, systemic treatment with bisphosphonates achieved a response.^[3]^ Indeed, bisphosphonates have been shown to induce apoptosis of GCT-ST cells in a dose-dependent manner in vitro.^[14]^ In addition, bisphosphonates can be an option for treating inoperable GCT-ST, although it is not included in the bone GCT therapy recommended by NCCN.^[13]^ Denosumab, a monoclonal antibody against RANK-L, is a targeted drug for unresectable bone GCT.^[13]^ Zoledronic acid and dinosemide systemic therapy have become available options for unresectable tumors.^[3]^ Interferon-α has been recommended to treat bone GCT,^[13]^ but its application in GCT-ST has not been reported.

RT can be used for unresectable or recurrent bone GCT, and radiation has the possibility of granulomatous transformation. Postoperative RT is recommended to prevent local recurrence and palliate the incomplete resection of the tumor. It has been reported that GCT-ST has radiosensitivity,^[15]^ but there is no literature report on RT for uterine GCT-ST.^[3]^ Although there is no detailed research to support postoperative RT, given its benign biology, the adjuvant RT of GCT-ST is controversial.^[1]^ When the margins are positive, and the adjacent structures allow it, RT can be considered.

In conclusion, GCT-ST is a rare tumor, especially in the uterus, pelvis, and abdomen. The clinical course of GCT-ST is usually benign, but it can display unpredictable biological characteristics. The take-home message of the case reported here is to ensure the complete removal of a GCT-ST with negative margins, including when the GCT-ST is an incidental finding in a non-oncological initial context. Adjuvant chemotherapy was given, but its actual efficacy is unknown.

## Author contributions

**Conceptualization:** Yao Jiang, Kaiyan Chen, Juan Qin.

**Data curation:** Yao Jiang, Kaiyan Chen.

**Formal analysis:** Mengting Yu, Juntao Wang.

**Methodology:** Mengting Yu.

**Writing – original draft:** Yao Jiang, Kaiyan Chen, juan Qin, Juntao Wang.

**Writing – review & editing:** Juan Qin, Juntao Wang.
